# Book Reviews

**Published:** 1822-10

**Authors:** 


### MONTHLY
### MEDICAL AND SCIENTIFIC BIBLIOGRAPHY.

Vexat censura ...... CORVOS.

ART. I.—*The Seats and Causes of Diseases investigated by Anatomy, &c.* By JOHN BAPTIST MORGAGNI. Abridged and elucidated with copious Notes, by WILLIAM COOKE, Member of the Royal College of Surgeons, and one of the Secretaries to the Hunterian Society.

THE various theories of physic excite admiration for a time, and are forgotten with the professors by whom they have been taught: even remedies, of which effects scarcely less than miraculous are recorded, pass away with the fashion which gave rise to their fame; but the authentic histories of diseased appearances remain permanent sources of knowledge, to which we can always turn for instruction, and never turn in vain. Such is the work of Morgagni. Our readers need not be afraid; we are not about to take upon ourselves a task of such supererogation as to give an account of that author's elaborate work, but merely to recommend to their notice a new translation of it. In a very unaffected and well-written preface, the author sets forth the object of the work. He shortly alludes to the importance of morbid anatomy, and the advantages which have accrued to medicine from its cultivation, and then follows a short biographical account of Valsalva and his illustrious pupil. Morgagni was born at Forli in Romagna, in 1682; he studied at Bologna, and attracted the notice of Valsalva and Albertini by his assiduity and talent. After having lectured for the former during his temporary absence, he proceeded to Venice, and thence to Padua, in the pursuit of professional acquirements. In 1711 he was appointed professor of the theory of medicine at Padua, and in 1715 chief professor of anatomy. The first part of his work was published in 1706, and afterwards continued at different times. In 1769 a translation was published in this country, by BENJAMIN ALEXANDER.

The obvious faults of the works of Morgagni, are their extreme bulkiness, their want of arrangement, and the desultory manner in which they are written: to obviate these inconveniences, is the object of the work before us. Mr. COOKE does not profess to have given a complete translation, but to have abridged and elucidated the work of Morgagni. He arranges the subjects under different sections; such as, "Phrenitis and Delirium," "Diseases of the Membranes of the Brain," "Hydrocephalus," &c.; and so far, it will be perceived, he has followed the general plan of the original. Under these sections are arranged all the cases which properly belong to them, culled from the different parts of Morgagni, by which means all diseases of the same parts, or morbid changes arising from the same disease, are placed together. In the last chapter are included "Fevers, Tumors, Diseases and Injuries of Bones and Joints, Strangulation, and Poisoning:" not, as must be very obvious, from any affinity between these subjects, but, as he informs us in a note, because, being residuary articles, he thought it better to associate them than to multiply divisions. Lastly, besides this improved arrangement, he has omitted all extraneous matter, of which the original contains too large a proportion, and condensed the whole. Such is the plan; of the manner in which it has been executed, our readers may form a judgment from the following specimen. We shall take the first case in Mr. Cooke's translation, because it is short, and give the text from the folio edition of Morgagni, published at Venice in 1761, Liber i. Epist. vii. art. 15.

"Mulier ex ictu capitis jam pridem in Patavino Nosocomeo decubuerat, et sana facta, discesserat. Postea in febrem incidit; deliravit; mortua est.

"Capite tantummodo, sub finem publicæ anatomes, quam anno habebam 1736 in gymnasium illato cerebrum a me sua in sede diligenter consectum. Nullum usquam peculiare illius ictus indicium. Crassa meninx sublata interiorem faciem ostendit crebris coccineis maculis quasi guttis sanguinis distinctam. Tenuis autem vasa sanguine turgida; sub eaque alicubi serum; quod in ventriculis non fuit. In plexuum choroidum posteriore parte vesiculæ. Ante glandulum pinealem subflavæ materiæ pauxillum. Cætera sana; nisi quod cerebellum fuit laxissimum. Nullo in vase quidquam polyposæ concretionis deprehendi."

Mr. Cooke's translation:

"A woman, who had long been in the hospital at Padua, in consequence of injury from a blow on the head, was dismissed cured. She was afterward attacked with fever, became delirious, and died.

*Dissection.*—There was no particular mark of the blow. The internal surface of the dura mater exhibited numerous scarlet spots, like drops of blood; the vessels of the pia mater were turgid, and serum was deposited beneath that membrane. There were vesicles in the posterior part of the choroid plexuses; the cerebellum was somewhat flaccid."

It will be perceived that the first sentence of the original, being quite unimportant, is very properly omitted altogether; next, that Mr. Cooke gives us only the positive, not the negative, information: he tells us that there was serum under the pia mater, but the "quod in ventriculis non fuit" of the original is not noticed; neither is the last sentence, "nullo in vase quidquam polyposæ concretionis deprehendi." It cannot be supposed that we have read all Mr. Cooke's book, but we have read and compared with the text several chapters, in different parts of each volume, and we think the one we have selected on account of its brevity gives a fair specimen of the manner in which the others are executed. As, however, we are "nothing if not critical," we would suggest that, in the chapter before us, "cerebellum fuit laxissimum" would have been quite as concisely, and rather more accurately, translated, very lax, as "somewhat lax;" and in chapter xxv. art. 6, in speaking of the nobleman who "in his youth had often contracted gonorrhœa and chancres," we find that the expression in the original is "gonorrhœis sæpe et penis ulceribus." Now it would, perhaps, have been better to give it literally: chancre is too specific a translation of ulcers in these times, when we hear every day of ulcers on the penis which are not chancres.

A part of the work which we must not pass over is the notes: these are in general good, and some of them very interesting; but they are, in many instances, too long, particularly those containing quotations. We regret this, because we suspect that notes, even if short, are apt to be disregarded, and we would not have so much information as these contain run the hazard of being overlooked. Would it not be more adviseable to give us an additional chapter, or to introduce them in the text, with the authorities from which they are taken; they would then claim the attention which they merit.

We must now take leave of Mr. Cooke and his translation. To have an edition of Morgagni, in which the extraneous matter was omitted, and the whole rendered accessible by improved arrangement, was a desideratum which all who have ever consulted his works must have experienced. This has been accomplished in the one before us; and we conclude by recommending it to all who would be saved unnecessary trouble in hunting over the original from one chapter to another, and more especially to those who, either from taste or *other motives*, find a good English translation more pleasant reading than the original Latin of Morgagni.

ART. II.—*Nouvelles Reserches et Observations sur la* GALE, *faites à l'Hopital St. Louis, à la Clinique de* M. LUGOL, *pendant les années* 1819, 1820, 1821; *et recueillies par* J. F. J. MOURONVAL, Docteur en Medicine de la Faculté de Paris. Avec 9 figures lithographièes. 1 vol. in 8vo.

IT is not surprising, amidst the revolutions and changes to which every thing in nature is liable, that few medical opinions should be found to be uncontroverted or incontrovertible. No articles of professional belief appear hitherto to have rested upon a firmer foundation than those respecting the existence of the itch insect, *(acarus scabiei,)* and the rapid and certain propagation of the disease by contact.

In the above work, however, we find both these positions denied, and a quantity of evidence adduced which *seems* satisfactorily to refute the first of these opinions, and very much to diminish the impression usually entertained by medical men, as well as the world at large, relative to the activity and virulence of the contagious property usually ascribed to this malady. M. Mouronval, as well as M. Lugol, claim our attention upon these points the more forcibly, as they appear to have been for some years firm believers in both the opinions above mentioned; and it is only in consequence of more extended observation and experience that they have been induced to abandon their original notions. We are informed by these gentlemen, that, in spite of the numberless researches they have made, with the best instruments, in all the forms and periods of the diseases, they have never been able to detect the insect in any one instance.

On turning to Dr. BATEMAN's work on Cutaneous Diseases, we find that he entertained great doubt as to the existence of the animalcula in *all* instances; and he quotes Dr. HEBERDEN, who not only never saw them himself, but who informs us that neither BAKER nor CANTON, who excelled in the use of the microscope, had ever been able to detect them: so far, therefore, we find that our authors have been anticipated in their doubts; and, though we are willing to concede to them that there is much room for deception, and much imagination often exercised in microscopic examinations, which, beyond a certain point, we conceive to be useless, and even illusory, yet we cannot understand the possibility of so many painful observers all agreeing upon a point, which we cannot conceive, after all, to be very difficult to decide; and not only asserting the existence of the acarus of the itch, but also giving drawings of its shape, number of legs, &c. &c. MOUFET, in England, was, we believe, the first person who described these animalculæ; but, since his time, CESTONI, REDI, MEAD, LINNÆUS, and MORGAGNI, have all added their testimony to that of our countryman; and one of these writers has declared that he has seen the insect deposit eggs. As late as the year 1812, M. GALES published a work on the Itch, in which he not only describes the insect, but succeeded in communicating the disease to himself and others, by placing these insects upon the back of his hand just before he went to bed, having previously warmed the part. The insects were kept in their situation by a watch-glass, fastened on with a bandage. The next day a strong sensation of itching was felt on the part, and three small miliary pustules were formed, and which being shown by him to several sçavans, among whom was M. RICHERAND, they were declared to be true itchy pustules. We need not continue our extract, but merely observe that the result of the inoculation of three children was still more conclusive. To recur once more to Dr. Bateman: it appears, that notwithstanding his quotation from Dr. Heberden, that he believed the acarus to exist in *some* instances, but was inclined to restrict it to a particular species; agreeing in that point with SAUVAGES, who speaks of a scabies vermicularis. Dr. B. farther declares, that though he had never detected it himself, he had seen it, in one instance, after it had been taken from the diseased surface by another practitioner.

We have thought it right to state these various evidences in contradiction to the opinion and experiments of M. Mouronval, not being prepared to decide on which side the truth may be found; but in the hope that this point may be finally established by the only test which can be applied to it, that of fair experiment.

Having decided upon the non existence of the acarus scabiei, our authors next proceed to inquire how the contagious property of the itch is produced. The experiments instituted by M. Mouronval show that he has inoculated himself with the matter from the pustules at different times; and that, finally, M. Lugol himself and a great many young medical men, together with Madame Fulgence, (religeuse de la Salle Ste. Marie,) were inoculated, either by the insertion of the matter or by rubbing it on those parts of the body where the eruption most commonly makes its appearance. This great number of persons proves, by the bye, only to have been nineteen. They were all carefully examined on the ninth day, and not one had taken the disease: a pimple was discovered in one instance upon one of the inoculated points, but it disappeared, without any remedy having been used, in about five or six days. M. Mouronval concludes, from these experiments, (a little too hastily, we think,) that the itch is not contagious. We wish that writers would draw no other conclusions from their facts but such as those facts fully bear out and warrant; and we are of opinion that the most that can be said to be actually proved by these trials, is that the itch is not *readily*, or perhaps at all, to be communicated by the insertion or rubbing-on of the matter into or upon the skin; but surely we cannot doubt, from the every-day experience of all army and navy surgeons, as well as from that of all persons attached to large institutions, that the itch is most certainly caught from sleeping with a patient infected with the disease, as well as by lying in infected sheets or using the body linen of those labouring under the complaint: M. Mouronval has, therefore, only succeeded in throwing doubts upon the *manner* in which the itch is propagated, and left us to conjecture that some particular condition of the skin may, perhaps, be requisite to render inoculation successful.

From all that we have seen, however, of this disease, we do not hesitate to declare that we do not think its contagious property is by any means so active as it is commonly supposed to be; and that, excepting when the body is warmed by the heat of the bed, or under other circumstances favourable for its development, it is very possible to come frequently in contact with persons affected by the disease, without receiving the infection. M. Mouronval distinguishes three forms of itch, the *batoneuse*, the *pustuleuse*, and the *miliare:* we think Dr. WILLAN's division into four species more just; but, as we are no sticklers for these distinctions, we will not insist upon this point. M. Mouronval gives ample directions for the treatment of these forms of the disease, but there is nothing particularly novel or striking in what he says; sulphur, in the form of vapour, baths, or ointment, being principally to be relied on.

---

